# Supplementary material for: Gaps in cancer care in a multi-ethnic population in Sarawak, Borneo: A central referral centre study
Source: PLoS One. 2024 Jul 26;19(7):e0296954. doi: 10.1371/journal.pone.0296954 (PMC11280152; doi:10.1371/journal.pone.0296954)
Supplement: Attachment — Submitted filename: Response to Reviewers.docx [file pone.0296954.s003.docx]

08.11.2023

Rebuttal Letter

Dear Reviewers and PLoS One Editorial team,

Thank you for taking time to review our manuscript and for your comments and feedback which we have incorporated in order to improve the manuscript. Please see below the rebuttal of each point raised in the revision comments.

Data Availability Statement

Upon re-submitting your revised manuscript, please upload your study’s minimal underlying data set as either Supporting Information files or to a stable, public repository and include the relevant URLs, DOIs, or accession numbers within your revised cover letter. For a list of acceptable repositories, please see http://journals.plos.org/plosone/s/data-availability#loc-recommended-repositories. Any potentially identifying patient information must be fully anonymized.

Important: If there are ethical or legal restrictions to sharing your data publicly, please explain these restrictions in detail. Please see our guidelines for more information on what we consider unacceptable restrictions to publicly sharing data.

Rebuttal: We attach the minimal data set as an SPSS data file with this submission.

Reviewer #1: This manuscript reports important summary information from a survey focusing on information provided to cancer patients undergoing follow up at the Department of Radiotherapy, Oncology and Palliative Care of the Sarawak General Hospital (SGH) in the state of Sarawak on the island of Borneo in East Malaysia. It has a number of strengths, including a large and diverse sample of participants from an under-studied region, a comprehensive survey about information provision (and participant satisfaction with this), and an assessment of potential discrepancies between self-reported importance of and satisfaction with a number of areas of information provision as part of their cancer care. In spite of these strengths, there are a number of weaknesses that reduce the potential impact of this work.

**1) The survey was designed to assess participant satisfaction with, and perceived importance of, a number of aspects relating to the cancer care they were receiving. However, the summaries that are provided for these questions do not appear to address the topic suggested by the title "Gaps in Cancer Care".**

Suggested Titles:

1.Gaps in Cancer Care in Sarawak, Borneo: A central referral centre study

2.The information needs and perception of cancer care in Sarawak, Borneo: A central referral centre study

3.Patients’ perception and satisfaction with cancer care in Sarawak, Borneo

4.Unmet information needs for cancer care in Sarawak, Borneo: A single tertiary centre study

5.Sarawak cancer patients’ unmet information needs: A central referral centre study

Rebuttal: Thank you, we have amended the title to the suggested ‘Gaps in Cancer Care in Sarawak, Borneo: A central referral centre study.’

**2) When summaries are provided for different domains, drastic differences in sample sizes are apparent. It is not completely clear from the methods what leads to these differences (though a partial description is provided in the statistical analysis section). If non-response is purely driven by participants' choices, these differences are informative to what might be concluded from the findings. More attention should be given to this, both from a view focusing on the description of the methods, as well as from a view about the potential for informative non-response. This could provide key and novel insights (e.g. the use of Western-driven domains in a different, non-Western context).**

Rebuttal: To address this – a column for correlation coefficient was added in Table 3. The perceived reason for the differences in sample size for certain domains such as sexual aspects are due to cultural barriers - as some patients are shy discussing sexual aspects and some patients may perceive this domain as being irrelevant due to age etc. We also made this domain optional due to the cultural sensitives surrounding sexuality. For the pain domain, this may be a gap in care as only a few patients were offered pain management as part of their cancer care, despite a high proportion of patients with advanced disease.

**3) It would have been informative had the demographic (and clinical) characteristics of the study participants been compared to reference values (e.g. all cancer patients seen at the medical center, all patients receiving care at the medical center, the population of the state).**

| **Type of Cancer** | **2020** | | | **2021** | | | **2022** | | |
| --- | --- | --- | --- | --- | --- | --- | --- | --- | --- |
|  | **Male** | **Female** | **Total** | **Male** | **Female** | **Total** | **Male** | **Female** | **Total** |
| **Breast** | 2 | 428 | **430** | 3 | 360 | **363** | 2 | 467 | **469** |
| **Lung** | 150 | 107 | **257** | 179 | 104 | **283** | 215 | 133 | **348** |
| **Colon** | 106 | 71 | **177** | 101 | 77 | **178** | 99 | 81 | **180** |
| **NPC** | 119 | 44 | **163** | 106 | 46 | **152** | 137 | 54 | **191** |
| **Cervix** | 0 | 138 | **138** | 0 | 109 | **109** | 0 | 165 | **165** |
| **Rectum** | 71 | 53 | **124** | 79 | 41 | **120** | 96 | 72 | **168** |
| **Stomach** | 46 | 27 | **73** | 56 | 29 | **85** | 42 | 28 | **70** |
| **Prostate** | 100 | 0 | **100** | 97 | 0 | **97** | 111 | 0 | **111** |
| **Ovary** | 0 | 72 | **72** | - | - | **0** | 0 | 78 | **78** |
| **Endometrium** | 0 | 68 | **68** | 0 | 72 | **72** | 0 | 91 | **91** |
| **Others** | 122 | 91 | **213** | 114 | 78 | **192** | 123 | 107 | **230** |
| **Liver** | - | - | **0** | 54 | 21 | **75** | 81 | 29 | **110** |
|  |  |  |  |  |  |  |  |  |  |

Rebuttal: Above are the statistics according to cancer types taken from Radiotherapy and Oncology Treatment Unit, Sarawak General Hospital – we do not have any data according to ethnicity as we do not have an electronic record system in the at Sarawak General Hospital at

**4) There are a number of methodological aspects of the manuscript that would benefit from additional information. These include:
a - More information might have been provided relating to the development and validation of the survey. Reporting a range of Chronbach's alpha values for the different (domain-specific?) internal consistencies is not adequate to enable careful assessment.**

**Questionnaire development**

The entire process of developing the questionnaire involved a total of five phases, as follows:

(i) to explore and understand the subject matter,
(ii) to develop the questionnaire,
(iii) to assess both its content validity and face validity,
(iv) to conduct a pilot study
(v) to undertake fieldwork using the questionnaire (Bujang & Tan-Hui, 2022). We began with a literature review of the published studies related to cancer care. Upon identification of a list of core components of cancer care, the authors then proceeded to develop the items that were further categorized into ten different domains including Diagnosis, Surgery, Radiotherapy, Systemic therapy, Pain management, Treatment monitoring, Psychosocial support, Sexual aspects, Financial, aspects and Overall experience. The overall aim of the questionnaire was to measure the perceived importance and overall satisfaction of cancer care amongst patients. An interval scale that was based on a five-point Likert scale, i.e., “not important/satisfied”, “slightly important/satisfied”, “neutral”, “important/satisfied” and “very important/satisfied”, was used in this study.

**Content Validity and Face Validity**

The content validity of the questionnaire was assessed by three subject matter experts including two senior physicians and a senior pharmacist. All the comments were given due consideration by the researcher, who made further amendments to the questionnaire in order to maintain content validity and also the overall accuracy of the content of each question/item. This was an iterative process, and was repeated several times until the overall results were found to be satisfactory. All the questions were initially developed in English. Subsequently, all the questions were translated into the Malay language by using both forward and backward translations. The study group carefully reviewed both the English and Malay versions of the questionnaire, and after making all necessary revisions for both versions of the questionnaire, an assessment of its face validity amongst three patients was conducted.

**Pilot study and fieldwork**

Once the results based on the assessment of both content validity and face validity were determined to be satisfactory, a pilot study was conducted amongst 10 patients. The aim of the pilot study was to test the reliability of the questionnaire. The Cronbach’s alpha of the domains ranged from 0.618 to 0.883. Following the pilot study, the final questionnaire to measure cancer care was developed with ten domains and 42 items.

*References*

*Bujang, M.A.; Tan-Hui, S. Development of Questionnaires for Quantitative Medical Research; Institute for Clinical Research, National Institute of Health, Ministry of Health Malaysia: Shah Alam, Selangor, Malaysia, 2022.*

**b - The reported sample size determination does not appear to align with the analyses that are actually reported in this manuscript.**

We have addressed this in the updated methods section.

Sample size statement

The sample size statement follows a guideline introduced by a previous study (Bujang, 2021). This study aims to determine the response from patients based on a survey of cancer care. The questions are measured based on a five point Likert-scale and thus the response was analysed in a numerical form, with mean (SD) reported. As the score for each item and domain are presented in terms of mean (SD), the sample size calculation is based on a formula to determine a mean for a targeted population. With an estimated standard deviation of 1.5 and a margin of error of 0.2, the minimum sample size requirement for a 95% confidence interval was calculated as 217 patients. Allowing for a 20.0% refusal/drop-out rate, the required sample size calculated was 272 patients.

The correlation test was also conducted between domain importance and domain satisfaction. Thus, sample size requirements also needed to be estimated based the Pearson’s correlation test. This calculation was based on the correlation coefficient = 0.0 in the null hypothesis versus a minimum correlation coefficient = 0.3 in the alternative hypothesis. Based on a minimum power of 80.0% and type I error of 5.0%, the minimum sample size requirement was 84 patients (Bujang and Baharum, 2016). To incorporate a 20.0% drop-out, the required sample size was 105 patients. Since the sample size requirement to estimate the mean for a population was higher, the target sample size requirement for this study was 272 patients.

*References*

*Bujang, M.A. A step-by-step process on sample size determination for medical research. Malays. J. Med. Sci. 2021, 28, 15–27.*

*Bujang MA, Nurakmal B. Sample size guideline for correlation analysis. World Journal of Social Science Research. 2016;3(1):37–46. doi: 10.22158/wjssr.v3n1p37.*

**c - It is not clear to this reviewer that a paired t-test comparing "importance" and "satisfaction" scores for specific questions (and domain averages?) is ideal for assessing the degree of agreement between (or differences in) these two areas of interest. Such an approach requires multiple assumptions (including those about comparability of measurement scales), and it is not clear that these were adequately considered or assessed.**

The analysis was changed to Pear.on’s correlation and updated under methods and table 3

A Spearman’s rank correlation test was applied to determine the correlation between response in both item and domain for importance and satisfaction since the assumption for the parametric test was violated.

(a change of t-test to Spearman’s rank correlation test was performed and changed in Table 3)

**d - Subset analyses are reported in the results, but descriptions of the approaches used were not provided.**

We have updated the methods and results section. Based on the correlation between importance and satisfaction in all domains, all correlations are significant with positive correlation (p<0.05) except for pain management (p=0.357), psychosocial support (p=0.260), sexual aspect (p=0.240), and financial (p=0.057). The correlations were not significant due to low correlation coefficients and/or a very small sample size for analysis in certain domains.

**5) There are a number of reporting practices that could be improved. Some examples include:**
**a - Summary values for comparisons of interest are often reported without a corresponding measure of variability (see e.g. 177, 178-179, 181, and many more reported comparisons).**

This has been amended in the manuscript.

**b - Figure 1 draws lines connecting the various domains together, yet there is no clear rationale as to why it might make sense to place them on a continuum. Removal of the smoothed lines is likely the most appropriate option here.
c - Figure 1 does not provide measures of variability for the reported "importance" and "satisfaction" means (or, perhaps more appropriately, for the differences between the two means).**

We have discussed this with our biostatistician and decided to remove Figure 1 from this manuscript and added the column for correlation coefficient in Table 3.

Reviewer #2: **1. Development of questionaries mentioned about self administrative. So the authors needs to explain the educational characteristics of respondents in methodology section. As you mentioned the study population is from diverse cultures. Please explain about how the self reporting survey is administrated?**

The questionnaire was administered by a research assistant, as stated in page 4 Line 109. The methodology has been updated accordingly.

The questionnaire was administred in 2 languages – Bahasa Malaysia (BM) and English. A Research Assistant approached patients waiting for their follow-up at RTU SGH and asked them if they wished to participate in a needs and perception survey. Patient who preferred to be interviewed in English will have their consent taken in English and those in BM will be in BM before the survey is conducted. As there were than 40 items being asked, this RA-assisted data collection is to ensure that every domain is being covered in the survey.

**2. The research topic is reflected disparity of cancer care of multi ethnic population in Sarawak. So the data needs to presents details disparity/difference of cancer care gaps within various ethnic population of Sarawak.**

Suggested title change incorporated as per reviewer 1.

**3. Discussion should be structured properly and should be more specific to the study. The authors should also discuss what the results of their study imply and cite and discuss more of relevant sources/articles.**

We have added some relevant articles to the discussion.

**4. Please highlight the key inference of the present study in conclusion section.**

We have highlighted some key references.

**5. At the end of the discussion, the limitations and suggestions for future studies should be mentioned separately.**

We have added a limitation section in the manuscript.

Reviewer 3

The draft is a well written approach on an important research topic. It addresses the gaps in cancer care of the multi-ethnic population of Sarawak, Borneo. The main research question is in which areas the holistic information provided to cancer patients by health care professionals needs strengthening.

Major issues

1.The research question is addressed via the comparison of the importance and satisfaction of the information provided by health care professionals. The approach is to calculate the difference of Likert scale rated items of importance and satisfaction for each item on a self- developed questionnaire. The definition of gaps in information delivery is defined as a statistically significant positive difference between the perceived importance and the satisfaction of the information provided.

Please state how you arrived at this definition. Is there any literature that supports this definition? To me this does not seem compelling. How do you ensure the person taking the questionnaire has the same underlying assumptions about satisfaction and importance and the distance between the single categories of ratings (which would be the starting point of arguing that the two items can be compared to identify unmet needs and, if this is not ensured, paired testing cannot be performed.)? Did you test both domains with the same Likert scale ratings as stated in the methods section? To scale satisfaction with the labels stated in the draft seems unusual, the intuitive way would be “very satisfied”, “satisfied” etc.

Rebuttal: **Questionnaire development**

The entire process of developing the questionnaire involved a total of five phases, as follows:

(i) to explore and understand the subject matter,
(ii) to develop the questionnaire,
(iii) to assess both its content validity and face validity,
(iv) to conduct a pilot study
(v) to undertake fieldwork using the questionnaire (Bujang & Tan-Hui, 2022). We began with a literature review of the published studies related to cancer care. Upon identification of a list of core components of cancer care, the authors then proceeded to develop the items that were further categorized into ten different domains including Diagnosis, Surgery, Radiotherapy, Systemic therapy, Pain management, Treatment monitoring, Psychosocial support, Sexual aspects, Financial, aspects and Overall experience. The overall aim of the questionnaire was to measure the perceived importance and overall satisfaction of cancer care amongst patients. An interval scale that was based on a five-point Likert scale, i.e., “not important/satisfied”, “slightly important/satisfied”, “neutral”, “important/satisfied” and “very important/satisfied”, was used in this study.

**Content Validity and Face Validity**

The content validity of the questionnaire was assessed by three subject matter experts including two senior physicians and a senior pharmacist. All the comments were given due consideration by the researcher, who made further amendments to the questionnaire in order to maintain content validity and also the overall accuracy of the content of each question/item. This was an iterative process, and was repeated several times until the overall results were found to be satisfactory. All the questions were initially developed in English. Subsequently, all the questions were translated into the Malay language by using both forward and backward translations. The study group carefully reviewed both the English and Malay versions of the questionnaire, and after making all necessary revisions for both versions of the questionnaire, an assessment of its face validity amongst three patients was conducted.

**Pilot study and fieldwork**

Once the results based on the assessment of both content validity and face validity were determined to be satisfactory, a pilot study was conducted amongst 10 patients. The aim of the pilot study was to test the reliability of the questionnaire. The Cronbach’s alpha of the domains ranged from 0.618 to 0.883. Following the pilot study, the final questionnaire to measure cancer care was developed with ten domains and 42 items.

*References*

*Bujang, M.A.; Tan-Hui, S. Development of Questionnaires for Quantitative Medical Research; Institute for Clinical Research, National Institute of Health, Ministry of Health Malaysia: Shah Alam, Selangor, Malaysia, 2022.*

*Another closely related research reference:*

*Chua GP, Tan HK, Gandhi M. What information do cancer patients want and how well are their needs being met?. Ecancermedicalscience. 2018;12:873. Published 2018 Sep 25. doi:10.3332/ecancer.2018.873*

2.How did you ensure that the differences in between the ratings of one scale alone (either satisfaction or importance) are assumed as equidistant (which would be starting assumption to treat the data as interval-scaled)? Al0s, how do you interpret -aside from the above given definition- a statistically significant difference of .367 (as in “You were given written information about the type of cancer you had”), considering that this does not even lead to a shift in the scaling given? Both items would be scored as “important” with a difference less than .5 which would be the minimal difference to lead to a shift in the rating. What would be the interpretation of a statistically significant negative difference? Or did you test one-sided? If so, please state this in the methods section.
Please also make sure you use the correct use of mean vs. difference in means (e.g., as in line 182)

Rebuttal:

The sample size statement follows a guideline introduced by a previous study (Bujang, 2021). This study aims to determine the response from patients based on a survey of cancer care. The questions are measured based on a five point Likert-scale and thus the response was analysed in a numerical form, with mean (SD) reported. As the score for each item and domain are presented in terms of mean (SD), the sample size calculation is based on a formula to determine a mean for a targeted population. With an estimated standard deviation of 1.5 and a margin of error of 0.2, the minimum sample size requirement for a 95% confidence interval was calculated as 217 patients. Allowing for a 20.0% refusal/drop-out rate, the required sample size calculated was 272 patients.

The correlation test was also conducted between domain importance and domain satisfaction. Thus, sample size requirements also needed to be estimated based the Pearson’s correlation test. This calculation was based on the correlation coefficient = 0.0 in the null hypothesis versus a minimum correlation coefficient = 0.3 in the alternative hypothesis. Based on a minimum power of 80.0% and type I error of 5.0%, the minimum sample size requirement was 84 patients (Bujang and Baharum, 2016). To incorporate a 20.0% drop-out, the required sample size was 105 patients. Since the sample size requirement to estimate the mean for a population was higher, the target sample size requirement for this study was 272 patients.

*References*

*Bujang, M.A. A step-by-step process on sample size determination for medical research. Malays. J. Med. Sci. 2021, 28, 15–27.*

*Bujang MA, Nurakmal B. Sample size guideline for correlation analysis. World Journal of Social Science Research. 2016;3(1):37–46. doi: 10.22158/wjssr.v3n1p37.*

3.Due to the problematic interpretation of those findings please state how you arrived at the assumption that the data is normally distributed and therefore can be evaluated with a paired t-test. Did you test for normal distribution? If so, please state that in the methods section. Also please be aware that the domain “pain management” has only 9 questionnaires completely filled in, therefore testing for normal distribution must be omitted since the number of cases to test for normality is too small. Likert scales may be treated as quasi-metric but only if summed up. In this publication however, all the single items are t-tested.

Rebuttal: A Spearman’s rank correlation test was applied to determine the correlation between response in both item and domain for importance and satisfaction since the assumption for the parametric test was violated.

(a change of t-test to Spearman’s rank correlation test was performed and changed in Table 3)

4.Since only 11 individuals answered some of the pain related questions, I was wondering why under three percent of the participants would reply – even more since it is stated in the methods section that the questionnaires were administered researcher assisted. Did you take any measures to assure no researcher bias would occur? Plus, in the discussion section is stated that only 11 people received pain management therapy. Were these the same 11 individuals answering the questions? Or is that coincidence? Or were the other participants just not offered this kind of treatment? Or just not asked whether they had gotten information about pain treatment?

Rebuttal: The 11 people who received pain management therapy in the discussion section are the same 11 individuals answering the questions. The other patients were not offered this kind of treatment and hence were not able to rate their ‘satisfaction’ or ‘importance’ opinion for this domain. These patients never had their pain assessed and would answer as ‘I don’t know’ when asked about the importance of pain management to be incorporated in their cancer care. Hence, those who never had pain killer or pain assessment during their cancer care journey were not asked further for this domain.

5.All these questions hint towards a major issue that is under-addressed in this paper: possible sources of bias, how this was accounted for while planning the study and while discussing the data (a crosstab of age vs gender could give an idea why family planning might have low response rates). Due to the massive amount of missings in some domains, an analysis of missing data would be expected. Is it at random?

Rebuttal: Most missing data are to address the gap in cancer care in our tertiary referral centre – mainly for non-diagnostic and treatment domain such as “Pain management” and “Financial care”. For the pain domain, those who were never on pain management were not asked further and for the financial care domain, missing data simply means that the patients does not understand the questions asked – such as “You were asked to check the availability of medical insurance coverage by your employer” – some are not employed or some does not know what does insurance means. To avoid bias in interpretation for this particular item, we will skip the rating for this item. However, for the “sexual aspects of care and family planning” domain, those who are of menopausal age and those who refuse to answer this due to the sensitive nature of this topic will not be asked further. Malaysia is a highly conservative country and some may interpret this domain negatively. However, this domain must not be omitted from their routine cancer care.

6.Considering the language/multiple ethics of the study population: was the questionnaire administered in the same language as the information given by health professionals? Might language be source of bias or not being satisfied with the information provided due to not understanding the information given? Could missings be attributed to this fact? Are not answered questions missings in terms of “this question does not apply to me”? Or maybe “I can ́t answer this question since this information was not given to me”? Or is it this a compound distribution of the above? Please make sure to include this information if you have it since it helps to get a feeling for possible bias. If participants had the possibility to state a non-applicability of an item or a domain, please do include this information also in the tabs.

Rebuttal: Yes considering the multiple ethnic and language of the study population, the gap in language given by healthcare professionals may be a source of bias. The non answered questions were missing in terms of “this question does not apply to me” Or even “I can ́t answer this question since this information was not given to me” or even “I don’t know because I don’t understand the context”. 80% of the respondents had only secondary school education and 7.9% had no formal schooling completed. Education barrier may also be another reason why some items were not comprehended by the respondents.

Please also go through the STROBE checklist (Lancet. 2007 Oct 20;370(9596):1453-7. PMID: 18064739) and make sure every item from this checklist is included in the publication. Some of the crucial points are stated in the above questions.

Please have the assistance of a biometrician/statistician to revise this draft if you have not had that until now.

Rebuttal: Table 3 has been revised by a biometrician/statistician according to his published guidelines:

1. *Bujang, M.A. A step-by-step process on sample size determination for medical research. Malays. J. Med. Sci. 2021, 28, 15–27.*
2. *Bujang MA, Nurakmal B. Sample size guideline for correlation analysis. World Journal of Social Science Research. 2016;3(1):37–46. doi: 10.22158/wjssr.v3n1p37.*
3. *Bujang, M.A.; Tan-Hui, S. Development of Questionnaires for Quantitative Medical Research; Institute for Clinical Research, National Institute of Health, Ministry of Health Malaysia: Shah Alam, Selangor, Malaysia, 2022.*

Minor issues

7.Please make sure -especially in the abstract- that the sentences are not too long. A flow chart would be nice but is not a necessity.

Rebuttal: We have edited.

8.Did you have the Malay questionnaire re-translated to English before administering it to the participants? If so, please state this in the methods section. This would help to gauge a language bias.

Rebuttal: The questionnaire was initially designed in English and then translated to Malay before being translated back to English (backward and forward translation) as stated in the methods section.

9.I could not completely reproduce the power calculation without making assumptions. Please make sure to include all the information necessary. Also please state whether and if so, how you have accounted for multiple testing If not please state that the results are of explorative nature.

Rebuttal:

Sample Size Statement

The sample size statement follows a guideline introduced by a previous study (Bujang, 2021). This study aims to determine the response from patients based on a survey of cancer care. The questions are measured based on a five point Likert-scale and thus the response was analysed in a numerical form, with mean (SD) reported. As the score for each item and domain are presented in terms of mean (SD), the sample size calculation is based on a formula to determine a mean for a targeted population. With an estimated standard deviation of 1.5 and a margin of error of 0.2, the minimum sample size requirement for a 95% confidence interval was calculated as 217 patients. Allowing for a 20.0% refusal/drop-out rate, the required sample size calculated was 272 patients.

The correlation test was also conducted between domain importance and domain satisfaction. Thus, sample size requirements also needed to be estimated based the Spearman’s rank correlation test. This calculation was based on the correlation coefficient = 0.0 in the null hypothesis versus a minimum correlation coefficient = 0.3 in the alternative hypothesis. Based on a minimum power of 80.0% and type I error of 5.0%, the minimum sample size requirement was 84 patients (Bujang and Baharum, 2016). To incorporate a 20.0% drop-out, the required sample size was 105 patients. Since the sample size requirement to estimate the mean for a population was higher, the target sample size requirement for this study was 272 patients.

*References*

*Bujang, M.A. A step-by-step process on sample size determination for medical research. Malays. J. Med. Sci. 2021, 28, 15–27.*

*Bujang MA, Nurakmal B. Sample size guideline for correlation analysis. World Journal of Social Science Research. 2016;3(1):37–46. doi: 10.22158/wjssr.v3n1p37.*

Many thanks.

Your sincerely

(Dr Yolanda Augustin)

Clinical Research Fellow

Global Oncology

St George’s University of London
